# Impact of organised colorectal cancer screening on age-specific population incidences: evidence from a quasi-experimental study in Sweden

**DOI:** 10.1007/s10654-023-01073-6

**Published:** 2024-01-04

**Authors:** Gabriella Chauca Strand, Ulf Strömberg, Anna Forsberg, Carl Bonander

**Affiliations:** 1 School of Public Health and Community Medicine, Institute of Medicine, Sahlgrenska Academy at University of Gothenburg, PO Box 469, 405 30 Gothenburg, Sweden; 2 Department of Medicine K2, Solna, 171 76 Stockholm, Karolinska Institutet Sweden

**Keywords:** Bowel cancer, Real-world evidence, Quasi-experimental, Synthetic control, Public health

## Abstract

Colorectal cancer (CRC) incurs a significant disease burden globally. Organised CRC screening programmes have been widely implemented for early detection and prevention. To understand the public health impact of these programmes, quantitative evidence of changes in overall and age-specific population incidences is fundamental. We aimed to provide such evidence by exploiting a time lag in the implementation of organised screening in Sweden: two out of 21 regions (these two regions comprise nearly 20% of the total Swedish population) have offered organised screening since 2008; the other regions have offered CRC screening since 2021. Using registry data on diagnosed CRC cases and socio-demographics for all regions in Sweden over the period 1970–2019, Bayesian structural time series modelling and difference-in-differences were applied to analyse the impact of screening on age-specific population incidences over time (CRC cases per 100.000 persons/year). After inviting birth-year cohorts aged 60–69 years for stool-based testing, the incidence rate in the 70–74-year age group decreased significantly over time, with an average reduction of − 44·40 (95% CI − 58·15 to − 31·31) from 2011 to 2019 in the intervention regions. In the overall population aged 60–74 years, there was a net incidence decrease of − 7·99 (95% CI − 13·85 to − 2·39) since the initiation of organised screening in the intervention regions (2008–2019). Organised CRC screening for 60–69-year-olds generated a change in age-specific incidence patterns with a long-lasting incidence decrease in the 70–74-year-old population, implying reductions in the excess mortality and burden of the disease.

**Supplementary Information:**

The online version contains supplementary material available at 10.1007/s10654-023-01073-6.

## Introduction

Colorectal cancer (CRC) is the second leading cause of cancer-related deaths globally. Despite decreasing mortality over time [1, 2], the incidence of CRC remains among the highest across cancers, causing a significant impact on the global burden of disease [2]. Screening for CRC has long been recommended as a means for prevention and early detection. Various screening methods exist and different strategies concerning the organisation of screening are being used worldwide [3].

Previous population-based studies have mainly focused on the benefit of screening in reducing CRC-specific mortality and total CRC incidence. Several observational studies have been conducted showing significant effects from screening on incidence and mortality [4–6], while systematic reviews of randomized controlled trials (RCTs) on the most common screening methods [7, 8], and newly emerged trial results [9], have shown inconclusive evidence regarding the impact on mortality and incidence for stool-based tests and colonoscopy.

Colorectal cancer screening has potential long-term benefits that include reduced mortality and incidence rates in the population. However, it is crucial to note that there might be an initial increase in incidence among those eligible for screening before the expected reduction in mortality and incidence rates becomes evident as screened individuals grow older. While RCTs are the gold standard when evaluating interventions, establishing preventive effects on overall incidence and CRC-specific mortality can thus take considerable time to ensure in controlled studies [10, 11]. Relatively little elaboration has been made on the impact of screening on incidence patterns over time across different age groups. Changes in age-specific incidence of screening have previously been analysed in observational studies, but only descriptively and with limited control for confounding [12–18]. Given sharply rising population incidences of CRC up to ages over 75 and the close connection between age-at-detection and the excess mortality from CRC [19, 20], it is fundamental to provide rigorously controlled real-world evidence for a long-lasting reduction in the CRC incidence in older age groups attributed to organised screening.

The present study aimed to address this gap by analysing the effects of CRC screening using biennial stool-based screening and colonoscopy on the overall and age-specific incidences of CRC in Sweden. Using a quasi-experimental design and two different nationally established registry data sets, we exploit a time lag in the implementation of organised screening in the Swedish regions to estimate the impact of organised screening on population CRC incidence patterns over time and across age groups.

## Methods

### Screening programme in Stockholm–Gotland

In 2008–2009, the regions of Stockholm and Gotland, as the only two of the 21 regions in Sweden (these two regions comprise nearly 20% of the total Swedish population), started implementing an organised screening programme using biennial guaiac faecal occult blood tests (gFOBT). Individuals aged 60–69 (birth year cohorts from 1940) were gradually enrolled in the screening programme. Invitations were sent to two randomly selected birth-year cohorts every year, along with information about the screening, test instructions, and a pre-paid return envelope. Individuals with positive tests were invited to a colonoscopy examination at a local clinic [21]. New screening invitations were made every two years, irrespective of prior participation. Supplementary Figure S1 gives an overview of the gradual enrolment and invitation of specific birth year cohorts starting from 2008. By 2011, seven cohorts had been enrolled in the programme and the first of the invited birth cohorts (1940) progressed to the post-screening age interval (i.e., aged >  = 70 years).

 In 2015, the gFOBT was replaced by faecal immunochemical tests (FIT). Participation rates were estimated up to 64% during the first five years of screening in the regions of Stockholm–Gotland with around 86–92% compliance to colonoscopy following a positive test result [21]. In 2023, the participation rate of the programme had increased to 71% [22]. A national recommendation for organised screening was issued in 2014 with the rest of the health regions initiating screening in 2021, targeting individuals aged 60–74 years.

### Primary analysis based on registry data in 1970–2019

We obtained publicly available data from the Swedish Cancer Registry on all incident CRC cases (International Classification of Diseases 7th Revision [ICD-7] codes: 153, 154; tumour types: 096) from the registry’s initiation in 1970 until the year before the COVID-19 pandemic, 2019 [23, 24]. The Cancer Registry is a law-based registry in which all cancer cases in the nation are required to be registered. This registry provides open-access data on region, sex, year, and age at diagnosis which was used for the primary analysis. Data on population sizes for each region, year, and sociodemographic strata (sex and age) were retrieved from publicly available data from Statistics Sweden based on the Total Population Register [25].

#### Data

Our study population consists of 60–74-year-olds for each year and region over the entire study period 1970–2019 which ensured the inclusion of the birth cohorts eligible and exposed to organised screening and their progression across age groups over the study period (Supplementary Figure S1). Supplementary Fig. S1 depicts an overview of the gradual enrolment of birth cohorts eligible for the screening programme and was used as a basis for structuring the analysis periods. We defined the period 1970–2007, before the organised screening began in Stockholm, as the pre-screening period and 2008–2019 as the primary post-intervention period. When analysing age-specific incidences among 70–74-year-olds, we defined 2011 as the first intervention year to reflect when the first birth-year cohorts (i.e., 1940 birth-year cohort) exposed to organised screening in 2009 entered this age group (Supplementary Figure S1).

Our outcome was incident cases per 100.000 person-years in the study population (60–74 years), including age-specific rates within this group. For the primary analysis, we created an analytical dataset by counting the number of cases per region, year, and socio-demographic strata in terms of age group and sex from 1970 to 2019. This was done for all Swedish regions.

#### Statistical methods

Our primary analysis uses Bayesian structural time-series models (BSTS) to estimate the counterfactual outcome trajectories in Stockholm–Gotland. We apply the method proposed by Brodersen et al. [26] who apply BSTS with a spike-and-slab prior to automatically construct a synthetic control unit using a weighted combination of outcome trajectories in non-intervention regions. The approach gives rise to a posterior distribution for the impact by comparing the posterior distribution of the synthetic control outcomes to the observed outcomes in Stockholm–Gotland after the introduction of organised screening. We computed average and time-varying impact estimates in the form of incidence rate differences per 100.000 person-years, percentage differences, and the cumulative difference in the number of cases, compared to the synthetic control. To implement the method, we used the ‘CausalImpact’ package for R version 4.1.1 (R Core Team, Vienna, Austria) with default settings [26]. See Supplemental Technical Appendix for details.

### Secondary analysis based on registry data in 2003–2019

We obtained an additional dataset of CRC cases registered between 2003 and 2019 from the Swedish Colorectal Cancer Registry (SCRCR) which was used as the basis for a secondary analysis. The SCRCR is a separate national register that contains approximately 98–99% of all CRC cases in Sweden [27]. Based on personal identification numbers, Statistics Sweden also helped us link socio-demographic register data to cancer cases in SCRCR, including the region of residence, educational attainment (classified according to the number of school years: ≤ 9 school years [no more than compulsory education], 10–12 school years [secondary education], and > 12 school years [corresponding to some education at university level]), and country of birth (Nordic, other Western, non-Western [Eastern Europe, Asia, Africa, and South America] countries). Data on the population size of each strata-year-region cell was provided by Statistics Sweden based on the Total Population Register and the Longitudinal integrated database for health insurance and labour market studies [25, 28]. The Swedish Ethical Review authority approved the collection and use of the data from the quality register SCRCR for the purpose of this study (dnr [2018/782-32]).

#### Data

In the secondary analysis, we used the national quality registry SCRCR to adjust for additional socio-demographic covariates. The analytical dataset was structured similarly to the primary analysis yet was stratified over additional socio-demographic variables (educational attainment and country of birth) and covered the period from 2003 to 2019. The pre-intervention period for this dataset was thus defined as 2003–2007.

#### Statistical methods

Due to the relatively short pre-intervention period for this dataset, a difference-in-differences (DID) estimation was used to analyse these data. The DID method, which compares outcomes before and after an intervention to a concurrent control group, relies on pre-intervention data within regions to account for unobserved, time-invariant confounders and data on trends from the control regions to adjust for secular time trends [29]. For this analysis we coded all post-intervention observations in Stockholm and Gotland as treated using a binary treatment dummy with the rest of Sweden (all other regions) being defined as controls. We used a weighted linear regression including region, year, and stratum-specific covariates effects (sex, age, educational attainment, and country of birth) with population weights, based on the population size in each strata, region, and year, to account for the varying population sizes in our stratified dataset. To account for serial correlation and possible small-sample bias in the DID estimations, we relied on a recently developed heteroscedasticity-robust residual cluster bootstrap, which has been shown to have good statistical properties with few treated clusters [30]. The secondary analysis was performed in Stata version 17.1 (StataCorp, TX). See Supplemental Technical Appendix for details.

### Sensitivity analyses

To assess model quality and violations of modelling assumption (e.g., the parallel trend assumption required for valid DID estimation), we performed in-time placebo tests following Abadie et al. [31]. In the primary analysis, we shifted the intervention date back in time by the length of the post-intervention period to use as a negative control period. For the secondary analysis, using SCRCR data, which covered a short pre-intervention period, the intervention date was shifted to 2006 instead of 2008 to assess violations of the parallel trend assumption. We also performed additional negative control analyses on the CRC incidence of younger age groups (50–59-year-olds) not eligible for the screening programme for both the primary and secondary analysis. To analyse possible time-varying treatment effects over the post-intervention period in the secondary analysis, using the DID method, we performed analyses across different post-intervention periods (screening roll-out [2008–2013] and widespread screening [2014–2019]). Finally, we performed two additional analyses for the primary analysis: (1) coding the intervention year to 2008 for the age group 70–74 years and (2) including the years 2020–2021 from the Cancer Registry, which were excluded from the main analysis to avoid confounding from the COVID-19 pandemic and initiation of organised screening in other regions.

### Role of the funding source

The funders of the study had no role in study design, data collection, data analysis, data interpretation, or writing of the report.

## Results

A total of 100.623 incident CRC cases in the age group 60–74 years (total study population) were registered over the period 1970 to 2019 (149·5 per 100.000 person-years in the total period).

 Tables 1 and 2 contains descriptive outcome and population data for the primary and secondary analyses, respectively. Stockholm–Gotland had a slightly lower incidence rate than the rest of Sweden at baseline (142·3 vs. 145·2 per 100.000 person-years, Table 1). Based on the descriptive data over the full period 1970–2019, the differences in incidence rates were even more pronounced in 2014–2019, after the organised screening programme in Stockholm–Gotland had reached almost complete coverage (142·3 vs. 168·4 per 100.000 person-years, Table 1). Overall, the regions of Stockholm–Gotland and the rest of Sweden were similar in age and sex distributions (Table 1). Socioeconomic data linked to the SCRCR register (2003–2019) suggest, however, that Stockholm/Gotland had a higher share of the population with tertiary education and fewer individuals born in Nordic countries (Table 2).

**Table 1:** Descriptive data based on the Cancer Registry data (1970–2019) on the study population and incident CRC cases in Stockholm–Gotland and the rest of Sweden (control), before and after the introduction of organised screening in Stockholm–Gotland in 2008/2009

	Stockholm–Gotland			Rest of Sweden (control)		
	Pre-screening period 1970–2007	Screening roll-out 2008–2013	Widespread screening 2014–2019	Period 1970–2007	Period 2008–2013	Period 2014–2019
Incident cases, n (rate per 100.000 person-years)	12 152 (142⋅3)	2711 (149⋅4)	2833 (142⋅3)	57 208 (145⋅2)	12 225 (162⋅2)	13 494 (168⋅4)
Person-years, n	8 535 876	1 814 106	1 990 860	39 402 432	7 535 405	8 012 304
Population size, annual mean	224 628	302 351	331 810	1 036 906	1 255 901	1 335 384
Population characteristics						
Age distribution, %						
60–64 yrs	38⋅8	39⋅6	35⋅2	36⋅8	38⋅5	33⋅5
65–69 yrs	33⋅1	36⋅0	33⋅3	33⋅6	35⋅4	34⋅3
70–74 yrs	28⋅1	24⋅4	31⋅5	29⋅1	26⋅1	32⋅3
Women, %	54⋅9	51⋅8	51⋅5	52⋅1	50⋅4	50⋅3

**Table 2:** Descriptive data based on the Swedish Colorectal Cancer Registry data (2003–2019) on the study population and incident CRC cases in Stockholm–Gotland and the rest of Sweden (control), before and after the introduction of organised screening in Stockholm–Gotland in 2008/2009

	Stockholm–Gotland			Rest of Sweden (control)		
	Pre-screening period 2003–2007^a^	Screening roll-out 2008–2013	Widespread screening 2014–2019	Period 2003–2007^a^	Period 2008–2013	Period 2014–2019
Incident cases, n (rate per 100.000 person-years)	1797 (142⋅2)	2571 (141⋅7)	2685 (134⋅9)	8518 (156⋅5)	11 562 (153⋅4)	12 681 (158⋅3)
Person-years, n	1 263 655	1 814 106	1 990 860	5 441 196	7 535 405	8 012 304
Population characteristics						
Educational attainment, %						
Primary, < = 9 yrs	27⋅5	21⋅9	17⋅8	41⋅5	32⋅4	25⋅0
Secondary, > 9 yrs < = 12 yrs	39⋅7	40⋅3	40⋅1	38⋅2	42⋅3	45⋅3
Tertiary, > 12 yrs	30⋅8	36⋅3	40⋅8	19⋅2	24⋅2	28⋅8
Missing	2⋅0	1⋅5	1⋅2	1⋅2	1⋅0	0⋅9
Region of birth, %						
Nordic country	88⋅2	87⋅1	83⋅4	94⋅4	93⋅8	92⋅0
Other Western countries	3⋅9	3⋅2	2⋅8	1⋅7	1⋅6	1⋅4
Non-western country	7⋅9	9⋅7	13⋅8	3⋅9	4⋅6	6⋅6

^a^Over the period 2003-2006, data on colon cancers have been complemented from the Cancer Registry due to lack of registration of colon cancers in the Swedish Colorectal Cancer Registry (SCRCR) over the period

**Fig. 1:**
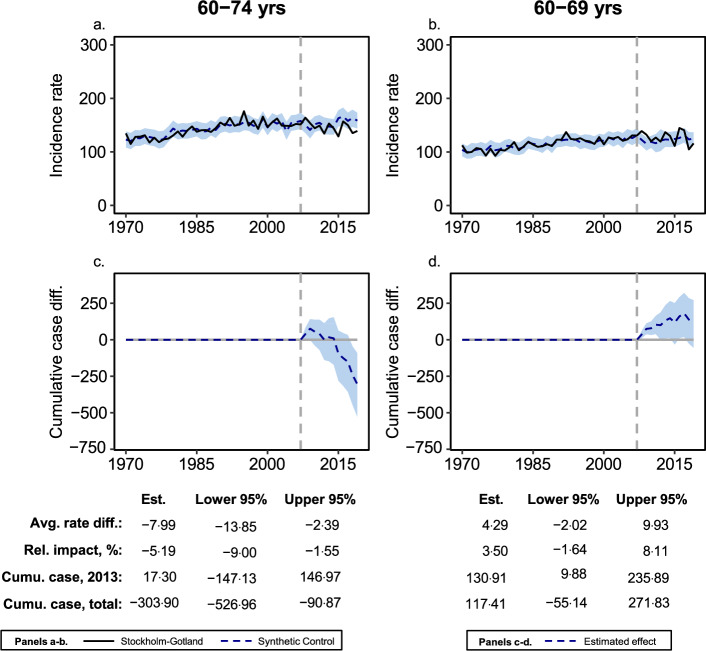
Overall CRC incidence trends and cumulative case differences over the period 1970–2019. Estimated rate differences, relative differences, and cumulative case differences with 95% CIs, are presented based on Bayesian structural time series analysis using the Cancer registry data. **a**, **b** Estimated trends of incidence rates for the total population (60–74) and screening eligible group (60–69) respectively. **c**, **d** Estimated cumulative differences in CRC cases between Stockholm–Gotland and the control regions for the total population (60–74) and the screening eligible age group (60–69) respectively

We found a − 7·99 (95% CI − 13·85 to − 2·39) reduction in CRC incidence per 100.000 person-years among 60–74-year-olds from 2008 to 2019 in Stockholm–Gotland compared to the synthetic control, corresponding to a 5% decrease in new CRC cases in the screened regions (Fig. 1). In total, the estimates suggest that the screening programme prevented 304 (95% CI − 527 to − 91) CRC cases among 60–74-year-olds in the intervention regions Stockholm–Gotland (mean population size of 320.000) over the course of 12 years after screening initiation.

**Fig. 2:**
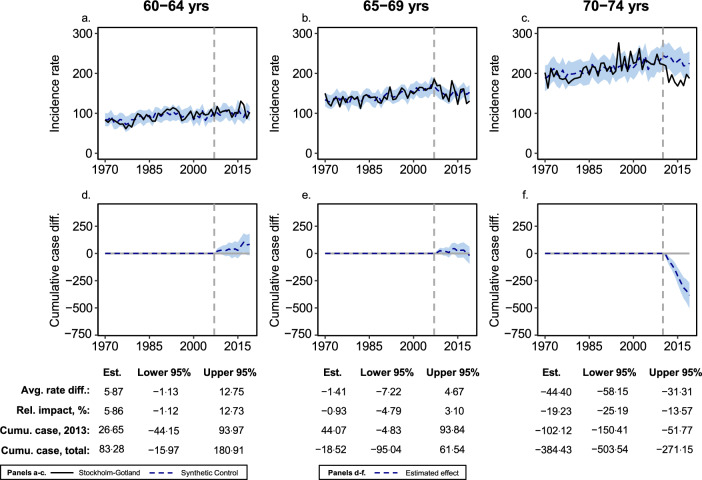
Age-specific CRC incidence trends and cumulative case differences over the period 1970–2019. Estimated rate differences, relative differences, and cumulative case differences with 95% CIs, are presented based on Bayesian structural time series analysis using the Cancer registry data. **a**–**c** Estimated trends of incidence rates for 5-year age groups 60–64, 65–69, and 70–74 years respectively. **d**–**f** Estimated cumulative differences in CRC cases between Stockholm–Gotland and the synthetic control for 5-year age groups 60–64, 65–69, and 70–74 years respectively

In the screening-eligible ages (60–69 years), our analysis suggests an initial increase in CRC incidence (Fig. 1). Stratified by five-year age groups, we found an average reduction of − 44·40 (95% CI − 58·15 to − 31·31) incident cases per 100.000 person-years among 70–74-year-olds in the period 2011–2019 (Fig. 2). The DID analysis using SCRCR data (2003–2019) showed a similar pattern of results. With this data, we could confirm that adjusting for observed sociodemographic differences does not influence the findings (Table 3). We also performed exploratory subgroup analyses to assess if the association between organised screening and CRC incidence differed across socio-demographic subpopulations (Supplementary Table S1). However, the only statistically significant effect modifier was age.

**Table 3:** Estimated impact of organised screening on incident CRC cases in Stockholm–Gotland using Difference-in-difference estimations based on CRC cases identified in the Swedish Colorectal Cancer Registry data (2003–2019)

	Age groups					
	60–74 years		60–69 years		70–74 years	
	Model (1)	Model (2)	Model (1)	Model (2)	Model (1)	Model (2)
Estimated rate difference						
Full period	− 3⋅48 (− 10⋅36, 3⋅40)	− 4⋅09 (− 10⋅92, 2⋅74)	1⋅43 (− 8⋅00,10⋅87)	2⋅20 (− 7⋅66,12⋅06)	− 22⋅56 (− 38⋅38, − 6⋅74)	− 20⋅69 (− 35⋅39, − 5⋅99)
	[0⋅321]	[0⋅240]	[0⋅766]	[0⋅662]	[0⋅005]	[0⋅006]
Screening roll-out	2·59 (− 6·75, 11·92)	1·28 (− 7·89, 10·45)	6·90 (− 3·80, 17·61)	6·77 (− 3·52, 17·06)	− 15·40 (− 36·28, 5·49)	− 14.96 (− 35·59, 5·66)
	[0 ·587]	[0·784]	[0·206]	[0·197]	[0·148]	[0·155]
Widespread screening	− 9⋅11 (− 18·08, − 0·14)	− 8·96 (− 17·57, − 0·35)	1·67 (− 8·91, 12·25)	2·76 (− 8·71, 14·23)	− 25⋅40 (− 42·97, − 7·83)	− 22⋅53 (− 39·67, − 5·39)
	[0·047]	[0·041]	[0·757]	[0·637]	[0·005]	[0·010]
Placebo test, 2006^a^	0·93 (− 11·90, 13·77)	0·86 (− 11·80, 13·51)	10·55 (− 4·06, 25·16)	10·39 (− 4·24, 25·03)	− 13⋅87 (− 45⋅12, 17⋅39)	− 13⋅73 (− 44⋅63, − 17⋅16)
	[0·887]	[0·895]	[0·157]	[0·164]	[0⋅385]	[0⋅384]

Rate differences expressed per 100.000 person-years. Confidence intervals (95% CIs) and *p*-values [in brackets] based on cluster-robust bootstrap with 5000 resamples (clustering by region). Estimates reflect the average impact over the full period 2008–2019 and post-intervention phases 2008–2013 and 2014–2019 respectively for the age groups 60–74 years (total study population) and 60–69 years. For the age group 70–74 years; estimates reflect the average impact over the period 2011–2019 (birth cohorts exposed to organised screening entered this age group in 2011, see Supplementary Fig. S1 for details) and post-intervention phases 2011–2013 and 2014–2019 respectively

(1) Difference-in-difference estimations including region and year fixed effects

(2) Difference-in-difference estimations adjusted for age groups (5-year groups), sex, educational attainment (primary, secondary, tertiary), and region of birth (Nordic, non-Western, other Western countries)

^a^In-time placebo test by restricting the data to the pre-intervention period and assigning a hypothetical intervention in 2006

In-time placebo tests showed no significant differences between Stockholm–Gotland and the rest of Sweden (Supplementary Figure S2). Coding the intervention year to 2008 for the group 70–74 showed a smaller estimate in the full period without affecting conclusions, − 41·53 (95% CI − 57·51 to − 25·80). Analyses including all publicly available data up until the year 2021 were similar to the main results (Supplementary Figure S3), and negative control analyses on 50–59-year-olds were non-significant and close to zero in both datasets and analysis methods (Supplementary Table S2).

## Discussion

In this study, we exploited a time lag in the implementation of an organised CRC screening programme in Sweden—where birth-year cohorts aged between 60 and 69 years were successively invited to biennial gFOBT/FIT testing starting in 2008 in Sweden—to assess the real-world impact of organised CRC screening on overall and age-specific incidences over time. We found a significant net reduction in overall CRC incidence in the total population aged 60–74 years over the period 2008–2019, and a more pronounced decrease in the incidence of CRC among 70–74-year-olds.

A strength of this study is that it is based on a quasi-experimental design and makes use of two nationally established registries covering all regions of Sweden. This allowed for a comprehensive comparison and follow-up to a concurrent control group over a long period, contrasting with previous studies of age-specific incidences [12–18]. The use of two different datasets including different covariates and analysis methods confirming the results is additionally a reassuring strength of the study.

The study also has limitations. Our study is quasi-experimental, and we cannot completely rule out other explanations for the observed results. However, in-time placebo tests and negative control analyses on younger age groups showed no strong evidence of bias. Spill-over effects, where changes in colonoscopies performed outside the organised screening programme have been observed over time [32], could be potential sources of bias. Likewise, the long study period could increase the possibility of changes in the composition between the treatment and control group due to e.g., migration, which can limit the identification of possible treatment effects. Our design relies on the assumption that equivalent developments over time in the intervention and control groups would have occurred in absence of the programme. Thus, in addition to controlling for all unobservable differences at baseline, the analysis can also handle unobserved confounders that would have developed similarly across the regions. However, bias due to differential changes in the post-intervention period that affect CRC incidences among 60–74-year-olds cannot be ruled out.

One possible confounding factor that we are aware of is the SCREESCO trial, which randomly enrolled persons aged 60–62 in organised screening as from 2014 [33], which may imply an underestimation of the impact of the organised screening programme in Stockholm–Gotland. Supplementary Figure S1 provides a description of the birth-year cohorts eligible for the SCREESCO trial—and the proportions enrolled in organised screening by the trial—in relation to the screening-exposed study population in Stockholm–Gotland considered in the present analysis.

Our finding of an overall reduction in CRC incidence among 60–74-year-olds indicates a direct preventive effect from the implementation of organised screening. The finding provides support for the hypothesis that organised screening induces extra removals of premalignant lesions that later manifest in a significantly long-lasting decline in the population incidence of CRC [34]. 

Previous studies on CRC screening have shown inconclusive evidence on the effects on overall incidence and mortality for stool-based tests [8], and recently colonoscopy [9]. Out of four trials evaluating gFOBT followed by colonoscopy, only one has shown statistically significant reductions in overall incidence after extensive follow-up [7, 8]. Likewise, trial results for primary colonoscopy showed unexpectedly low effects on overall incidence, and no evidence of a mortality reduction, contrary to previous studies [4, 9]. Considerations of participation, adenoma detection rates, and the need for longer follow-up times to establish effects have been discussed regarding the contrasting evidence on CRC incidence and mortality from screening trials [10, 11]. Our results provide real-world evidence of a reduction in the total CRC incidence up to 12 years after the implementation of organised screening with an average participation rate of approximately 64% [21]. 

We also observed changes in age-specific incidences and a long-lasting reduction in the incidence among 70–74-year-olds i.e., in an age stratum of the population following the screening age span. Observations of an increasing incidence followed by a reduction within the screening-eligible age groups (with varying age intervals from 50 up to 74 years) over time have been observed in previous studies [12–14, 16, 18]. The evaluated programme in this study targeted 60–69-year-olds and included a long follow-up which could explain the more pronounced decrease in incidence among the older age group 70–74 compared to previous findings [12–14]. Previous research on age-specific incidences after screening implementation has largely relied on incidence trend analyses or comparisons against historical controls. By contrast, our study was based on a quasi-experimental design, allowing for a long-term controlled evaluation against a concurrent control for all age groups. Thus, our results provide stronger evidence of causality regarding the impact of screening on age-specific incidences over time.

The observed reduction among 70–74-year-olds could have important implications on the burden of disease due to CRC. Apart from a preventive effect which is substantiated in this age group, the initial increase in incidence trends preceding the reduction among 70–74-year-olds, further indicates a shift in the detection of CRC, from older to younger age groups, due to screening. Age is a crucial factor for treatment possibilities, the probability of CRC survival, and the burden of disease [35]. In previous analyses of CRC excess mortality in Sweden, the estimated excess mortality due to CRC, was found to be 43% higher among the age group 70–74 years compared to those aged 55–69 years after adjustment with tumour stage at diagnosis [19]. Globally, significant differences in CRC survival have also been shown across different age groups [35].

Our results on age-specific incidences could thus indicate additional benefits in reducing excess mortality due to CRC and the burden of disease, which the sole evaluation of the effects on overall incidence with limited follow-up could obscure when estimating the benefits generated from screening.

In conclusion, our study provides real-world evidence that the implementation of organised screening for CRC in the regions of Stockholm–Gotland has generated a reduced overall incidence and a clear change in age-specific incidences, yielding decreasing incidence time trends among older age groups. Given the need to evaluate and understand the effectiveness of different programmes on population health, analysis of trends in age-specific incidences can further highlight the effect of screening in reducing the excess mortality and burden of the disease at a population level.

### Supplementary Information

Below is the link to the electronic supplementary material.

Supplementary file1 (DOCX 692 KB)

## Data Availability

The data from the Swedish Cancer Registry are publicly available and can be obtained in original form from the Swedish National Board of Health and Welfare. We have also posted the data and R code for this part of the study in a public repository available at https://osf.io/6c7ms/. The SCRCR data, on the other hand, cannot be shared publicly as the data contain potentially identifying and sensitive personal data according to Article 9 of the General Data Protection Regulation (EU 2016/679), and public availability would compromise participant privacy. The General Data Protection Regulation (EU 2016/679) also classifies de-identified versions of sensitive data that are sufficiently detailed to allow for re-identification of sensitive personal information. According to Swedish law (Law 2003:460 for ethical review of research involving humans), ethical permission is required to process such data. In accordance with Swedish legislation, the data can and will be made available to researchers who meet the criteria for access to confidential data, which includes obtaining their own ethics approval from the Swedish Ethical Review Authority (email: registrator@etikprovning.se; website: https://etikprovningsmyndigheten.se).
