# Correction: Impact of organised colorectal cancer screening on age-specific population incidences: evidence from a quasi-experimental study in Sweden

**DOI:** 10.1007/s10654-024-01115-7

**Published:** 2024-05-06

**Authors:** Gabriella Chauca Strand, Ulf Strömberg, Anna Forsberg, Carl Bonander

**Affiliations:** 1 School of Public Health and Community Medicine, Institute of Medicine, Sahlgrenska Academy at University of Gothenburg, PO Box 469, 405 30 Gothenburg, Sweden; 2 Department of Medicine K2, Solna Karolinska Institutet, 171 76 Stockholm, Sweden

## Correction to: European Journal of Epidemiology (2024) 39:87–96 10.1007/s10654-023-01073-6

In Table 2 of this article, the footnote was missing and should have read ‘Over the period 2003–2006, data on colon cancers have been complemented from the Cancer Registry due to lack of registration of colon cancers in the Swedish Colorectal Cancer Registry (SCRCR) over the period’.

**Table 2:** Descriptive data based on the Swedish Colorectal Cancer Registry data (2003–2019) on the study population and incident CRC cases in Stockholm–Gotland and the rest of Sweden (control), before and after the introduction of organised screening in Stockholm–Gotland in 2008/2009

	Stockholm–Gotland			Rest of Sweden (control)		
	Pre-screening period 2003–2007^a^	Screening roll-out period 2008–2013	Widespread screening period 2014–2019	Period 2003–2007^a^	Period 2008–2013	Period 2014–2019
*Outcome data*						
Incident cases, n (rate per 100.000 person-years)	1797 (142.2)	2571 (141.7)	2685 (134.9)	8518 (156.5)	11,562 (153.4)	12,681 (158.3)
Person-years, n	1,263,655	1,814,106	1,990,860	5,441,196	7,535,405	8,012,304
*Population characteristics*						
Educational attainment, %						
Pre-secondary, ≤ 9 years	27.5	21.9	17.8	41.5	32.4	25.0
Secondary, > 9 years ≤ 12 years	39.7	40.3	40.1	38.2	42.3	45.3
Post-secondary, > 12 years	30.8	36.3	40.8	19.2	24.2	28.8
Missing	2.0	1.5	1.2	1.2	1.0	0.9
Region of birth, %						
Nordic country	88.2	87.1	83.4	94.4	93.8	92.0
Other western country	3.9	3.2	2.8	1.7	1.6	1.4
Non-western country	7.9	9.7	13.8	3.9	4.6	6.6

^a^Over the period 2003–2006, data on colon cancers have been complemented from the Cancer Registry due to lack of registration of colon cancers in the Swedish Colorectal Cancer Registry (SCRCR) over the period

In Table 3 of this article, the ‘Screening roll-out’ data in the column ‘70–74 years’ was incorrectly published with – 9.03 (− 29.88, 11.81), − 8.83 (− 29.43, 11.77) [0.401] [0.396] and should have been − 15.40 (− 36.28, 5.49) [0.148], − 14.96 (− 35.59, 5.66) [0.155]

Incorrect version:

**Table:** 

	Age groups					
	60–74 years		60–69 years		70–74 years	
	Model (1)	Model (2)	Model (1)	Model (2)	Model (1)	Model (2)
Estimated rate difference						
Full period	− 3.48 (− 10.36, 3.40)	− 4.09 (− 10.92, 2.74)	1.43 (− 8.00, 10.87)	2.20 (− 7.66, 12.06)	− 22.56 (− 38.38, − 6.74)	− 20.69 (− 35.39, − 5.99)
	[0.321]	[0.240]	[0.766]	[0.662]	[0.005]	[0.006]
Screening roll-out	2.59 (− 6.75, 11.92)	1.28 (− 7.89, 10.45)	6.90 (− 3.80, 17.61)	6.77 (− 3.52, 17.06)	− 9.03 (− 29.88, 11.81)	− 8.83 (− 29.43, 11.77)
	[0.587]	[0.784]	[0.206]	[0.197]	[0.396]	[0.401]
Widespread screening	− 9.11 (− 18.08, − 0.14)	− 8.96 (− 17.57, − 0.35)	1.67 (− 8.91, 12.25)	2.76 (− 8.71, 14.23)	− 25.40 (− 42.97, − 7.83)	− 22.53 (− 39.67, − 5.39)
	[0.047]	[0.041]	[0.757]	[0.637]	[0.005]	[0.010]
Placebo test, 2006^a^	0.93 (− 11.90, 13.77)	0.86 (− 11.80, 13.51)	10.55 (− 4.06, 25.16)	10.39 (− 4.24, 25.03)	− 13.87 (− 45.12, 17.39)	− 13.73 (− 44.63, − 17.16)
	[0.887]	[0.895]	[0.157]	[0.164]	[0.385]	[0.384]

Rate differences expressed per 100.000 person-years. Confidence intervals (95% CIs) and *p* values [in brackets] based on cluster-robust bootstrap with 5000 resamples (clustering by region). Estimates reflect the average impact over the full period 2008–2019 and post-intervention phases 2008–2013 and 2014–2019 respectively for the age groups 60–74 years (total study population) and 60–69 years. For the age group 70–74 years; estimates reflect the average impact over the period 2011–2019 (birth cohorts exposed to organised screening entered this age group in 2011, see Supplementary Fig. 1 for details) and post-intervention phases 2011–2013 and 2014–2019 respectively

(1) Difference-in-difference estimations including region and year fixed effects

(2) Difference-in-difference estimations adjusted for age groups (5-year groups), sex, educational attainment (primary, secondary, tertiary), and region of birth (Nordic, non-Western, other Western countries)

^a^In-time placebo test by restricting the data to the pre-intervention period and assigning a hypothetical intervention in 2006

Corrected version:

**Table 3:** Estimated impact of organised screening on incident CRC cases in Stockholm–Gotland using difference-in-difference estimations based on CRC cases identified in the Swedish Colorectal Cancer Registry data (2003–2019)

	Age groups					
	60–74 years		60–69 years		70–74 years	
	Model (1)	Model (2)	Model (1)	Model (2)	Model (1)	Model (2)
Estimated rate difference						
Full period	− 3.48 (− 10.36, 3.40)	− 4.09 (− 10.92, 2.74)	1.43 (− 8.00, 10.87)	2.20 (− 7.66, 12.06)	− 22.56 (− 38.38, − 6.74)	− 20.69 (− 35.39, − 5.99)
	[0.321]	[0.240]	[0.766]	[0.662]	[0.005]	[0.006]
Screening roll-out	2.59 (− 6.75, 11.92)	1.28 (− 7.89, 10.45)	6.90 (− 3.80, 17.61)	6.77 (− 3.52, 17.06)	− 15.40 (− 36.28, 5.49)	− 14.96 (− 35.59, 5.66)
	[0.587]	[0.784]	[0.206]	[0.197]	[0.148]	[0.155]
Widespread screening	− 9.11 (− 18.08, − 0.14)	− 8.96 (− 17.57, − 0.35)	1.67 (− 8.91, 12.25)	2.76 (− 8.71, 14.23)	− 25.40 (− 42.97, − 7.83)	− 22.53 (− 39.67, − 5.39)
	[0.047]	[0.041]	[0.757]	[0.637]	[0.005]	[0.010]
Placebo test, 2006^a^	0.93 (− 11.90, 13.77)	0.86 (− 11.80, 13.51)	10.55 (− 4.06, 25.16)	10.39 (− 4.24, 25.03)	− 13.87 (− 45.12, 17.39)	− 13.73 (− 44.63, − 17.16)
	[0.887]	[0.895]	[0.157]	[0.164]	[0.385]	[0.384]

Rate differences expressed per 100.000 person-years. Confidence intervals (95% CIs) and *p* values [in brackets] based on cluster-robust bootstrap with 5000 resamples (clustering by region). Estimates reflect the average impact over the full period 2008–2019 and post-intervention phases 2008–2013 and 2014–2019 respectively for the age groups 60–74 years (total study population) and 60–69 years. For the age group 70–74 years; estimates reflect the average impact over the period 2011–2019 (birth cohorts exposed to organised screening entered this age group in 2011, see Supplementary Fig. 1 for details) and post-intervention phases 2011–2013 and 2014–2019 respectively

(1) Difference-in-difference estimations including region and year fixed effects

(2) Difference-in-difference estimations adjusted for age groups (5-year groups), sex, educational attainment (primary, secondary, tertiary), and region of birth (Nordic, non-Western, other Western countries)

^a^In-time placebo test by restricting the data to the pre-intervention period and assigning a hypothetical intervention in 2006

The original article has been corrected.

