# Supplementary material for: Impact of organised colorectal cancer screening on age-specific population incidences: evidence from a quasi-experimental study in Sweden
Source: Eur J Epidemiol. 2024 Jan 4;39(1):87–96. doi: 10.1007/s10654-023-01073-6 (PMC10810926; doi:10.1007/s10654-023-01073-6)
Supplement: Supplementary file 1 — Supplementary file1 (DOCX 692 KB) [file 10654_2023_1073_MOESM1_ESM.docx]

**Supplementary Material**

**European Journal of Epidemiology**

**Impact of organised colorectal cancer screening on age-specific population incidences: evidence from a quasi-experimental study in Sweden**

Gabriella Chauca Strand, Ulf Strömberg, Anna Forsberg, Carl Bonander

School of Public Health and Community Medicine, Institute of Medicine, Sahlgrenska Academy at University of Gothenburg, PO Box 469, SE-405 30 Gothenburg, Sweden (G Chauca Strand MPH, U Strömberg PhD, C Bonander PhD); Department of Medicine K2, Solna, Karolinska Institutet, SE-171 76 Stockholm, Sweden (A Forsberg MD PhD).

Table of contents

|  | Page |
| --- | --- |
| Figure S1 | 2 |
| Figure S2 | 3 |
| Figure S3 | 4 |
| Figure S4 | 5 |
| Table S1 | 6 |
| Table S2 | 7 |
| Technical Appendix | 8 |

**Figure S1.** Schematic overview of the implementation of organised screening for colorectal cancer in the Region of Stockholm-Gotland, Sweden, 2008-2019 and SCREESCO trial, 2014-2019.

| **Birth year:** |  |  |  |  |  |  |  |  |  |  |  |  |  |
| --- | --- | --- | --- | --- | --- | --- | --- | --- | --- | --- | --- | --- | --- |
| 1961 |  |  |  |  |  |  |  |  |  |  |  |  |  |
| 1960 |  |  |  |  |  |  |  |  |  |  |  | s |  |
| 1959 |  |  |  |  |  |  |  |  |  |  | s | **×** |  |
| 1958 |  |  |  |  |  |  |  |  |  | s | **×** | s |  |
| 1957 |  |  |  |  |  |  |  |  | s | **×** | s | **×** |  |
| 1956 |  |  |  |  |  |  |  | s | **×** | s | **×** | s |  |
| 1955 |  |  |  |  |  |  | s | **×** | s | **×** | s | **×** |  |
| 1954 |  |  |  |  |  |  | **×** | s | **×** | s | **×** |  |  |
| 1953 |  |  |  |  |  |  | s | **×** | s | **×** |  | **×** |  |
| 1952 |  |  |  |  | **×** |  | **×** | s | **×** |  | **×** |  |  |
| 1951 |  |  |  |  |  | **×** | s | **×** |  | **×** |  | **×** |  |
| 1950 |  |  | **×** |  | **×** |  | **×** |  | **×** |  | **×** |  |  |
| 1949 |  | **×** |  | **×** |  | **×** |  | **×** |  | **×** |  |  |  |
| 1948 |  |  |  |  | **×** |  | **×** |  | **×** |  |  |  |  |
| 1947 |  |  |  |  |  | **×** |  | **×** |  |  |  |  |  |
| 1946 | **×** |  | **×** |  | **×** |  | **×** |  |  |  |  |  |  |
| 1945 |  |  |  |  |  | **×** |  |  |  |  |  |  |  |
| 1944 |  | **×** |  | **×** |  | **×** |  |  |  |  |  |  |  |
| 1943 |  |  |  | **×** |  |  |  |  |  |  |  |  |  |
| 1942 | **×** |  | **×** |  |  |  |  |  |  |  |  |  |  |
| 1941 |  |  |  |  |  |  |  |  |  |  |  |  |  |
| 1940 |  | **×** |  |  |  |  |  |  |  |  |  |  |  |
| **Calendar year:** | 2008 | 2009 | 2010 | 2011 | 2012 | 2013 | 2014 | 2015 | 2016 | 2017 | 2018 | 2019 |  |
|  |  |  | | | | | | | | | | |  |
|  | Notes: Overview of the enrolment of birth-year cohorts colorectal cancer screening programme in the regions of Stockholm-Gotland. The birth-cohorts within the target population (60–69) were randomly invited and gradually enrolled to the programme. Colours indicate the evolvement across age-groups over time and was used as a basis for defining the post-screening periods of the analyses. | | | | | | | | | | | | |
|  |  | The persons of the given birth-year cohort aged ≤60 years in the given calendar year | | | | | | | | | | | |
|  |  | The persons of the given birth-year cohort aged between 60-65 years in the given calendar year | | | | | | | | | | | |
|  |  | The persons of the given birth-year cohort aged between 65-70 years in the given calendar year | | | | | | | | | | | |
|  |  | The persons of the given birth-year cohort aged between 70-75 years in the given calendar year | | | | | | | | | | | |
|  |  | The persons of the given birth-year cohort aged ≥75 years in the given calendar year | | | | | | | | | | | |
|  | **×** | The persons of the given birth-year cohort who lived in the intervention region were invited to stool-based testing (FOBT in 2008-2014, FIT in 2015-2019). N.B. Persons born in 1941 have not been invited.^[[1]](#footnote-1)^ | | | | | | | | | | | |
|  | s | The persons of the given birth-year cohort in the control regions who *may* have received an invitation to the SCREESCO trial at a specific year. The SCREESCO trial included 91 440 individuals within the ages of 59–62 over the period 2014–2019,^[[2]](#footnote-2)^ constituting around 25% of the total population of the control regions. Mean population size for each age-group over the period of recruitment, 90 000 individuals. The trial did not include the regions of Stockholm-Gotland. | | | | | | | | | | | |

**
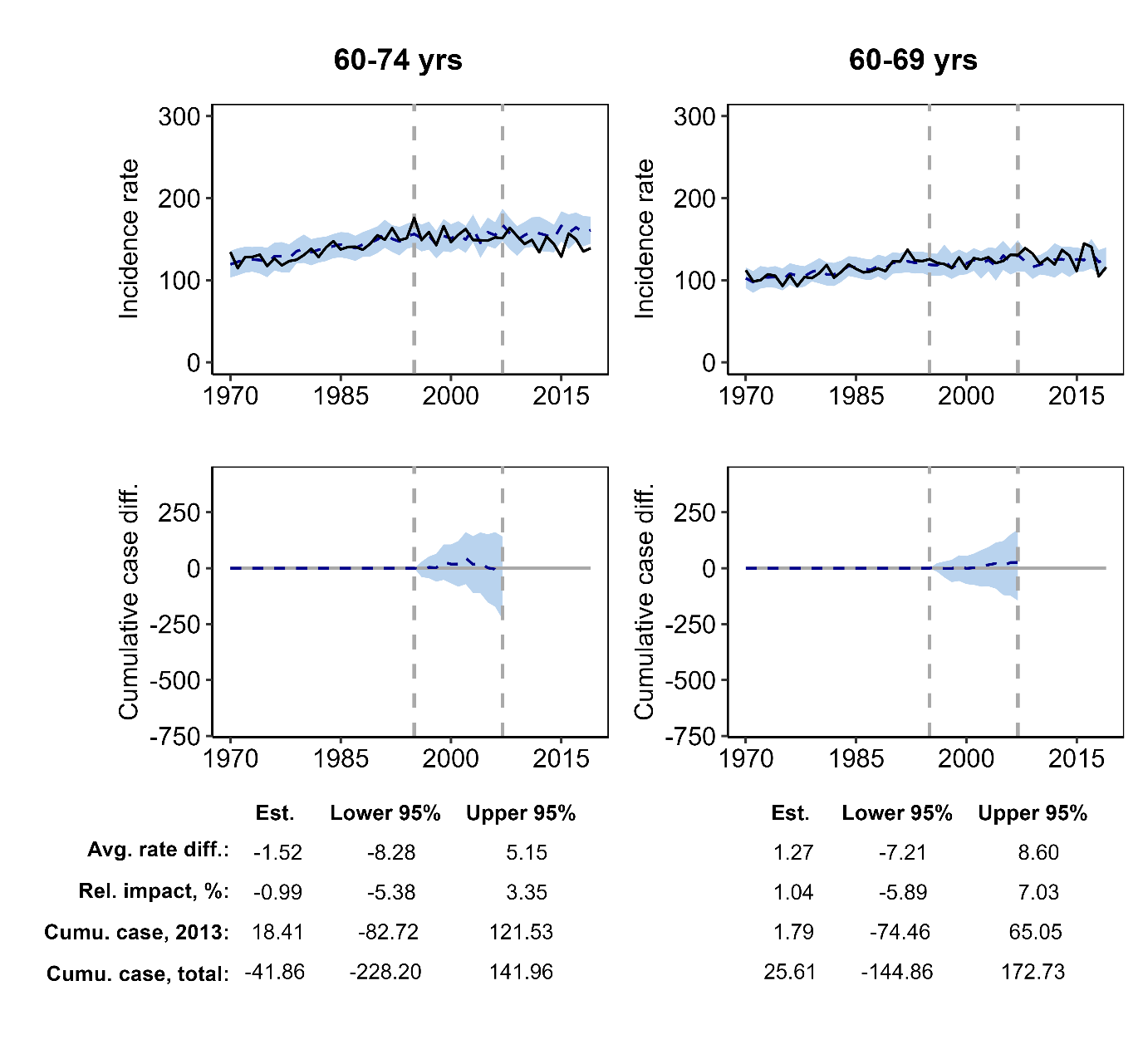
**

**Figure S2.** Results from in-time placebo analyses backdating the intervention by the length of the post-intervention period to analyse a hypothetical intervention occurring in the pre-intervention data for 60–74-year-olds and 60–69-year-olds respectively. Estimates are based on Bayesian structural time series analyses using data from the Cancer Registry (1970–2019), and the results show no evidence of impacts before the initiation of organised screening.

**
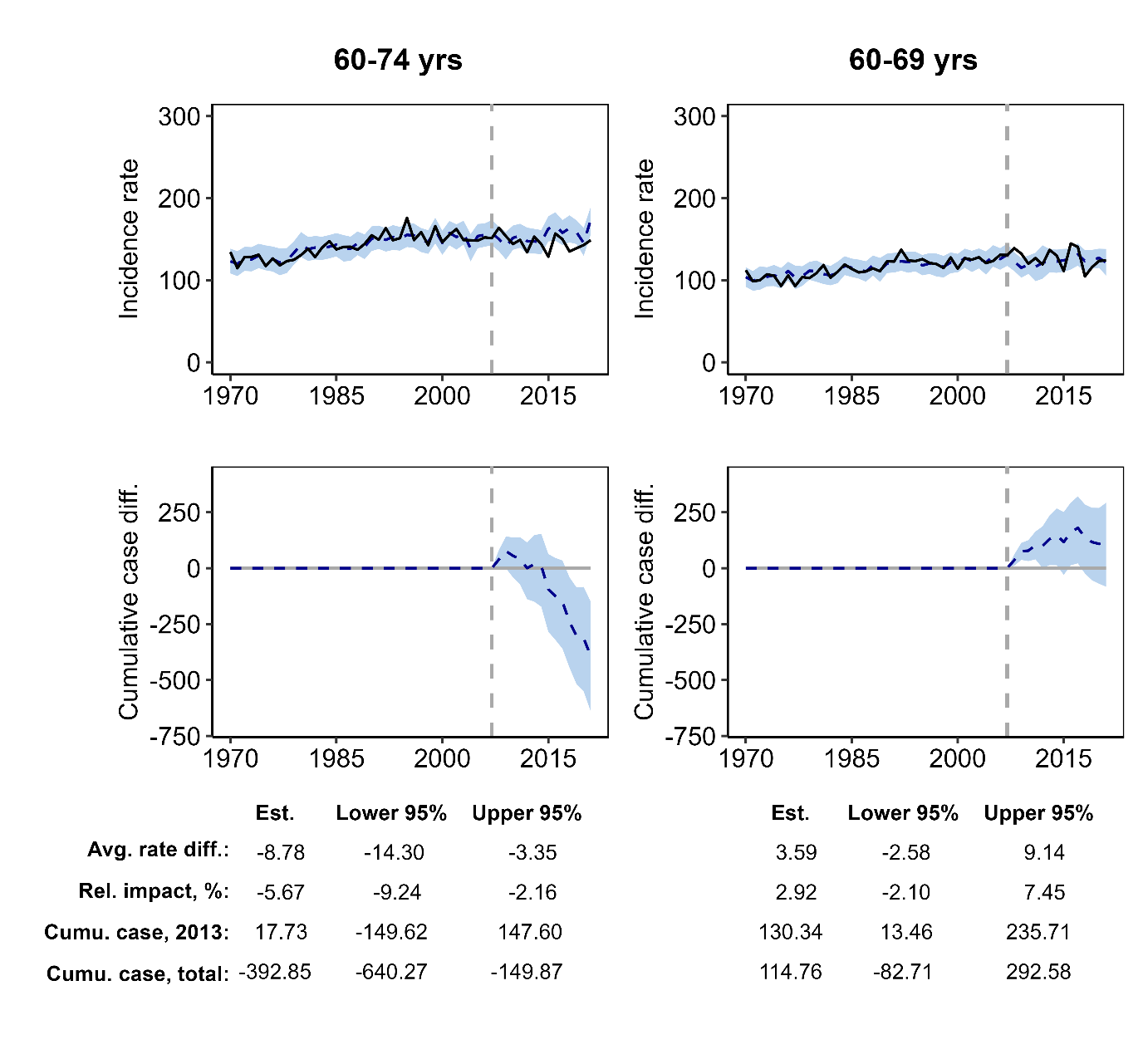
**

**Figure S3.** Estimated rate differences, relative effects, and cumulative case differences with 95% CI from the sensitivity analyses using a Bayesian structural time series approach and publicly available data (Cancer registry data) until 2021, for the total study population (60–74) and age group 60–69**.**  The upper figures display trends of colorectal cancer incidence between Stockholm-Gotland (solid line) and the synthetic control (dashed). The lower figures indicate the cumulative case differences between Stockholm-Gotland and the synthetic control.

**Figure S4.** Trends in colorectal cancer incidence per 100.000 person-years in the intervention regions Stockholm-Gotland and the rest of Sweden from 2003 to 2019. The plotted graphs are based on data from the Swedish Colorectal Cancer Registry (SCRCR) used for the DID estimation. The vertical dashed line indicates the start of the screening programme roll-out in Stockholm-Gotland in 2008.

| **Table S1. Association between organised screening in Stockholm-Gotland and incidence rate per 100.000 person-years by sociodemographic characteristics, 2003–2019.** | | | |  |
| --- | --- | --- | --- | --- |
|  | **DID estimate**  **(2008–2019)** | | **Placebo test**  **(2006)** |  |
| **5-year age-groups** | |  |  |  |
| 60–64 yrs | | 9⋅11 | 3⋅98 |  |
|  | | (0·41, 17⋅81) | (-11⋅97, 19⋅93) |  |
|  | | [0⋅040] | [0⋅625] |  |
| 65–69 yrs | | -5⋅46 | 6⋅67 |  |
|  | | (-20⋅69,9⋅77) | (-18⋅17, 31⋅51) |  |
|  | | [0⋅483] | [0⋅599] |  |
| 70–74 yrs | | -17⋅53 | -13⋅73 |  |
|  | | (-31⋅65, -3⋅40) | (-45⋅03, 17⋅56) |  |
|  | | [0⋅015] | [0⋅390] |  |
| **Sex** | |  |  |  |
| Men | | -4⋅90 | -4⋅29 |  |
|  | | (-14⋅86, 5⋅07) | (-25⋅34, 16⋅76) |  |
|  | | [0⋅336] | [0·689] |  |
| Women | | -3⋅20 | 5⋅37 |  |
|  | | (-12⋅24, 5·84) | (-12⋅23, 22⋅97) |  |
|  | | [0·488] | [0⋅550] |  |
| **Country of birth** | |  |  |  |
| Nordic | | -5⋅79 | -0⋅54 |  |
|  | | (-13⋅45, 1⋅88) | (-13⋅20, 12⋅12) |  |
|  | | [0⋅139] | [0⋅972] |  |
| non-Nordic | | 2⋅46 | -3⋅08 |  |
|  | | (-38⋅61, 43⋅52) | (-137⋅47, 131⋅31) |  |
|  | | [0⋅907] | [0⋅964] |  |
| **Educational attainment** | |  |  |  |
| Primary school | | -0⋅29 | 8⋅46 |  |
|  | | (-10⋅85, 10⋅26) | (-15⋅73, 32⋅65) |  |
|  | | [0⋅957] | [0⋅526] |  |
| Secondary school or higher | | -2⋅38 | -1⋅02 |  |
|  | | (-10⋅03, 5⋅27) | (-17⋅45, 15⋅42) |  |
|  | | [0⋅542] | [0⋅903] |  |
| *Notes:* Difference-in-difference estimates of the impact of organised screening stratified by sociodemographic characteristics using the Swedish Colorectal Cancer Registry (SCRCR) dataset. All models in the table include adjustments for region fixed effects, year fixed effects, age group, sex, educational attainment, and country of birth. The estimates reflect incidence rate differences per 100.000 person-years. Cluster-robust confidence intervals (95%) based on a cluster-robust residual bootstrap with 5000 resamples are shown in parentheses with p-values in square brackets. | | | | |

| **Table S2. Negative control analyses on 50–59-year-olds** | |
| --- | --- |
| **Analysis** | **Estimates** |
| Bayesian structural time series analysis^a^ | 1⋅98 |
|  | (-1⋅33, 4⋅93) |
| Difference-in-differences analysis^b^ | 0⋅35 |
|  | (-5⋅24, 5⋅94) |
| *Notes:*  Placebo analyses using unscreened age-groups 50–59 years as negative controls for both the primary and secondary analysis. Numbers reflect incidence rate differences per 100.000 person-years. Confidence intervals (95%) are shown in parentheses.  a Data from the Cancer Registry, 1970–2019.  b Data from the Swedish Colorectal Cancer Registry, 2003–2019. The model includes adjustment for region fixed effects, year fixed effects, age group, sex, educational attainment, and country of birth. | |
|  | |

# Technical appendix

## Notation

We use the potential outcomes framework [1] to clarify our causal estimands and models. In the following, $Y_{jt}(w)$ represents the potential outcome under intervention state $w\in\{0,1\}$, where $1$ means that region $j$ is exposed to the intervention at year $t$.

## Synthetic control analysis

Our synthetic control analysis relies on the ‘CausalImpact’ package for R [2], with default settings. The package fits a Bayesian structural time-series model to predict the counterfactual outcomes without organised screening, using the framework outlined in Brodersen et al [3]. Essentially, the model is trained on the pre-intervention data and then predicts the counterfactual untreated potential outcomes in Stockholm-Gotland in the post-intervention period. We opted for a Bayesian variant of the synthetic control method because it offers a coherent framework for uncertainty estimation. Bayesian statistics combine prior beliefs, which are often specified before seeing the data, with observed data to make inferences. In the case of ‘CausalImpact’, most of the priors are set based on pre-intervention data with an empirical Bayes approach [2]. Uncertainty is then quantified using posterior distributions, which arise from combining these priors with the observed data.

Bayesian structural time series models provide a structured way to analyse time series data through latent variables. In our model, there are two core components: the observation equation, which links the observed data to a latent local level, and the state equation, which dictates how this local level evolves over time. The observation equation captures deviations from the latent state, while the state equation models its inherent dynamics.

The observation level equation in the synthetic control model can be expressed as follows:

1. $Y_{1t}\left( 0 \right)=\mu_{t}+\sum_{j=2}^{N} \beta_{j}Y_{jt}\left( 0 \right)+\epsilon_{t},$

where:

- $Y_{1t}(0)$ is the untreated potential outcome at time *t* in the intervention region (*j=1*).
- $N$ is the number of regions in the data.
- $\mu_{t}$ is the local level at time *t*.
- $\beta_{j}$ is the synthetic control weight for control region *j*.
- $\epsilon_{t} \sim N(0,\sigma_{\epsilon}^{2})$ is the observation error, normally distributed with mean 0 and variance $\sigma_{\epsilon}^{2}$.

The state equation can be expressed as follows:

1. $\mu_{t+1}=\mu_{t}+\xi_{t},$

where $\xi_{t} \sim N(0,\sigma_{\xi}^{2}$) is the system error at time *t*. The state equation captures the progression of the local level over time. This progression is influenced by the level at time *t* and a system error or shock,$\xi_{t}$, which determines its direction and magnitude. The local level term models the inherent, unobserved trends in the treated region that are not reflected by changes in control outcomes, resembling a random walk influenced by noise. The predictive importance of this term largely hinges on the accuracy with which the synthetic control component of the model predicts the outcomes.

While most of the hyperparameters are estimated from the pre-intervention data using an empirical Bayes approach, the standard deviation for the local level must be set a priori [2]. We set the standard deviation of the local level in our model to 0.01, which is the package default. In essence, this choice reflects our prior assumption that the yearly outcomes should be stable with low volatility after regressing out the synthetic control outcomes [2].

In Equation (1), $\beta_{j}$ represents the unit weight of the $j^{th}$ control unit in the synthetic control. The weights are estimated using a spike-and-slab prior to encourage sparsity in the selection of controls (i.e., it favours simpler synthetic controls with fewer controls involved, which helps to control the risk of overfitting). See Brodersen et al [3] for details.

Once the model has been fit, we obtain the post-intervention predictions of the untreated (counterfactual) outcomes:

1. $\hat{Y}_{1t}\left( 0 \right)=\mu_{t}+\sum_{j=2}^{N} \beta_{j}Y_{jt}\left( 0 \right).$

The estimated intervention effect in Stockholm-Gotland at time *t* is then given by:

1. $\hat{\alpha}_{1t}=Y_{1t}-\hat{Y}_{1t}\left( 0 \right)$,

where $Y_{1t}$ is assumed, by counterfactual consistency, to represent the treated potential outcome $Y_{1t}(1)$ in post-intervention time points. Uncertainty is then quantified by sampling from the posterior distribution of the estimated effects using Markov Chain Monte Carlo estimation [4].

For a causal interpretation, the following assumptions must hold:

1. The synthetic control region is not influenced by the intervention (i.e., no spillovers).
2. The relationship between the control regions (i.e., the synthetic control weights) and the treated time series, established in the pre-intervention period, remains stable across time. This stability ensures the validity of predicting the counterfactual outcomes in the post-intervention period [2,3]. This implies that events uniquely affecting the treated region or synthetic control in the post-intervention period, such as concurrent interventions, should not occur. In essence, we assume that the effects of all such events are shared by both the treated unit and synthetic control.

## Difference-in-differences

Our difference-in-difference (DID) estimation relies on a standard linear regression framework [5]:

1. $Y_{jst}=\mu_{j}+\delta_{t}+\alpha D_{jt}+\mathbf{X}_{s}+\epsilon_{jst},$

where:

- $\mu_{j}$ are region fixed effects that capture all time-invariant region-level unobserved confounders.
- $\delta_{t}$ are time fixed effects, which capture unobserved common (national) trends.
- $\alpha$ is the difference-in-difference estimate of the effect.
- $D_{jt}$ is a binary treatment variable coded as 1 for observations in Stockholm-Gotland after the initiation of screening, and zero otherwise.
- $\mathbf{X}_{s}$ represent strata-specific covariates and their effects.

We fit this model on the entire dataset to estimate the average effects, and on subgroups represented of the sociodemographic variables in $\mathbf{X}_{s}$ to estimate subgroup-specific effects, $\alpha_{s}$. Observations used in the DID analyses reflect sociodemographic strata. To accommodate varying population sizes across strata, we assign weights to the DID models based on the region-strata-year-specific population size $P_{jst}$.

While the DID model parallels the synthetic control model, it operates under stricter assumptions. Specifically, the DID model necessitates the 'parallel trends assumption' [5], positing that in the absence of the organised screening intervention, the treated region and controls would have followed identical trends in colorectal cancer incidence.

The ’parallel trends assumption’ is inherently untestable as it involves the post-intervention counterfactuals, but it is often probed by checking if outcome trends are non-parallel in the pre-intervention data. To check for non-parallel trends in our data, we perform an in-time placebo test [6]. Using data from only from the period 2003–2007 (i.e., before the organised screening intervention), we fit the same DID model as in Equation (5), but re-code the $D_{jt}$ variable to 1 in 2006–2007 in Stockholm-Gotland. Essentially, this means that we test for intervention effects before any should occur. If the parallel trends assumption holds, we should expect the placebo effect estimate from this model to be negligible.

# References

1. Rubin DB. Causal Inference Using Potential Outcomes. J Am Stat Assoc. 2005;100:322–31.

2. Brodersen K, Gallusser F, Koehler J, Remy N, Scott SL. CausalImpact: An R package for causal inference in time series [Internet]. [cited 2023 Sep 17]. Available from: http://google.github.io/CausalImpact/

3. Brodersen KH, Gallusser F, Koehler J, Remy N, Scott SL. Inferring causal impact using Bayesian structural time-series models. Ann Appl Stat. 2015;9:247–74.

4. Hamra G, MacLehose R, Richardson D. Markov Chain Monte Carlo: an introduction for epidemiologists. Int J Epidemiol. 2013;42:627–34.

5. Angrist JD, Pischke J-S. Mostly Harmless Econometrics: An Empiricist’s Companion. Princeton University Press; 2009.

6. Abadie A, Diamond A, Hainmueller J. Comparative Politics and the Synthetic Control Method. Am J Polit Sci. 2015;59:495–510.

1. Strömberg U, Holmén A, Peterson S, Pålsson B. Geomapping av tjock- och ändtarmscancer. Lokalisation av geografiska områden för riktade informationskampanjer när nationell tjock- och ändtarmscancerscreening införs. Regional Cancer Centrum (RCC), 2022. [↑](#footnote-ref-1)
2. Forsberg, Anna et al. Once-only colonoscopy or two rounds of faecal immunochemical testing 2 years apart for colorectal cancer screening (SCREESCO): preliminary report of a randomised controlled trial. The Lancet Gastroenterology & Hepatology, Volume 7, Issue 6, 513 - 521 [↑](#footnote-ref-2)
